# President’s Address

**Published:** 1904-07-15

**Authors:** J. P. Beauman

**Affiliations:** Columbus, Ohio


### PRESIDENT'S ADDRESS.

BY J. P. BEAUMAN, D.D.S., COLUMBUS, OHIO.

Read before the Ohio State Dental Society, December, 1903, and printed from advance proofs by courtesy of the "Dental Summary."

Another year has passed into history, and once more we have assembled for the thirty-eighth session of the Ohio State Dental Society, which we have good reason to believe will be one of the best, and we hope the most profitable, meetings the society has ever held. We extend a most cordial greeting to all members and friends, and hope all may return to their homes feeling that they have been well repaid for being present. We most heartily wish every one to feel perfectly at home while here and free to take part in the program with us and help to make a success of this meeting.

The program that has been prepared and presented for your consideration we believe will meet with your hearty approval. The program and work in preparation for this meeting have cost the several committees much time and hard labor, and I freely accord to these committees all credit for the successful manner in which the work has been accomplished.

Complaints have come to our notice from members of the profession that little attention has been accorded them, and that they could not get acquainted. I would therefore charge the committee on membership, also the members in general, that if you see a man you do not know, take him by the hand, and make him feel that you want to know him; ask him his name, where he lives, and where he was born, how long he has been a dentist, etc., and if you find him eligible, get his application for membership.

We have in the State of Ohio about twenty-six hundred members of the dental profession, and out of this twentysix hundred we have less than three hundred active members of this society. Is it possible that we have so few men in the State who care for the elevation of the science of dentistry and the progressive work of this society?

This society, as well as that of all others, is truly an educational institution, and every one can receive much benefit by becoming an active member. I therefore recommend that the society at this session take some decided action, or formulate some plan whereby the eligible dentists of the State may be brought into close touch with the work of this society.

The second article in our constitution clearly defines the purpose and the object of this association, which should be upheld by every one who loves his profession and humanity. The profession has been making great advancement in the past, and to-day the work is onward and upward, and yet there are much greater fields to explore by the twentieth century dentist. Every man who wishes to keep pace with his profession in the twentieth century must make use of all the helps at his command, and there is none more efficient than to connect in a practical way in society work.

Commercialism has taken hold of the people in all walks of life to an alarming extent. So much so, that man has gone mad in pursuit of the almighty dollar. This same spirit prevails in dentistry as well as in all other occupations. The dentist who has lost sight of his higher obligations to humanity, and can see nothing but dollars, has become a mountebank in his profession and should be ostracised by all true and honorable professional men. Empiricism can not be driven from the field by legislative action and the enforcement of law. The public looks upon it as a line of persecution for selfish ends. The law may prescribe certain rules and require of the graduate a most rigid examination before a board, but when he has passed said examination and has received his diploma and certificate, he is then free to go forth and advertise all kinds of nostrums and practice all manner of deceptions upon a credulous public, and the law can not lay hands upon him for not complying with the code of ethics.

It is most desirable that all should work for a uniform code of laws in all the States of the Union, so that the man who has passed a creditable examination in one State may be entitled to equal privileges in every other State.

The law should guard the rights of the public as far as it is in the province of law; but there are other moral obligations resting on every man who enters a profession, that has to deal with the physical being (which is the most sacred to the individual of anything in life). He should, from professional pride, have no desire to mutilate the human body, in any manner whatever, for commercial or selfish gain. To purify the stream, you must purify the fountainhead from whence the stream flows. As long as the dental colleges are run in sharp competition on commercial lines, by sending agents out through the State and country, working up matriculates for their respective schools, giving rebates, etc., it will be slow progress in eliminating commercialism and empiricism from the profession. The way to break down empiricism is not by law, but by building up a higher standard of scholarship and dignity in the ethical relations in the profession.

Our present civilization demands more highly-educated and cultured men in the learned professions. The twentieth-century dentist must be a cultured and scholarly man. If the colleges are run on commercial lines, can you question the right of the dental graduate when he goes forth from the schools, to commence business by advertising his wares and conducting his practice in channels of commercialism? To rectify these errors is the work of this society, as well as of the profession at large. As we shall have a paper on our code of ethics, I will not dwell on the subject further.

There is one great responsibility resting on the profession, and one that should command most serious consideration, and that is the education of the public in general to a proper understanding of the care and treatment of the natural teeth. Parents should be most carefully instructed as to their responsibility in the care of their children's teeth, both temporary and permanent. I formulated a plan many years ago that would work if put into practice, but time and space will not permit of a delineation here.

It is an easy thing for the president in his annual address to make recommendations, but it is not so easy to get the society, as well as the profession, to put them into practice. I would exhort each and every one to pluck fruit from the topmost branches of the tree of science, as in the higher branches, the most perfect and beautiful fruit grows, where the sun shines and the light is unobstructed.

Since our last session we have lost by death two of our most worthy and prominent members, Drs. Otto Arnold and Jonathan Taft. We shall all feel this bereavement when we no more find these familiar faces with us. Both these men stood for the higher education for the dental profession. No man has spent more time and money in attending society meetings than Dr. J. Taft. He always stood ready to take part in reading papers, in the discussion of papers read, or to take part of committee work in any society. In 1860 or 1861, we organized what was called the Central Ohio Dental Society. The society held four or five annual meetings. Doctor Taft was with us at Zanesville, Delaware and Mt. Vernon meetings. At Mt. Vernon, steps were taken to organize the State Society. The charge was placed upon Dr. Taft to send out the call, which was done, and assembling was had in the old Naughton Hall. Dr. Taft took an active part in the organization of this society at that time. He has never missed in attendance since that time, but is now absent for the first time. All honor to the man who has been thus faithful in his obligations to the society he helped to organize.

I know of but two who helped in the organization of this society in 1861 who are living, and that is Dr. H. A.

Smith and myself. The years are passing and it will soon be left to others to carry the work forward until the depth of dental science has been explored. I would exhort each and every one to climb to the topmost rung of the ladder. Climb high. What you have to do, do it to-day.

